# Reporting of immune-related adverse events in US Food and Drug Administration approvals of immune checkpoint inhibitors

**DOI:** 10.3389/fonc.2025.1606599

**Published:** 2025-08-13

**Authors:** Shaili Tapiavala, Chongliang Luo, Mina Shenouda, Vaibhav Patel, Andrew A. Davis

**Affiliations:** ^1^ Division of Oncology, Washington University in St. Louis, St. Louis, MO, United States; ^2^ Division of Public Health Sciences, Washington University in St. Louis, St. Louis, MO, United States; ^3^ Division of Hematology and Medical Oncology, Tisch Cancer Institute, Icahn School of Medicine at Mt. Sinai, New York, NY, United States

**Keywords:** immuno-oncology, toxicity, immune-related adverse events, immune checkpoint inhibitors, trial reporting, morbidity

## Abstract

**Background:**

Predicting the occurrence of immune-related adverse events (irAEs) related to immune checkpoint inhibitors (ICI) is complex. Monitoring of irAEs is critical as toxicities cause morbidity and impact quality of life. Thus, we systematically evaluated the patterns and consistency of irAEs reporting in trials leading to US Food and Drug Administration (FDA) ICI approvals.

**Methods:**

We evaluated 75 primary articles from 2011-2021. The authors independently collected data regarding reporting frequency as a binary classification of reported versus not reported and irAE frequency of 24 irAEs classified by the National Comprehensive Cancer Network Version 1.2024 guidelines. Reporting trends and irAE events were analyzed by study year, phase, primary tumor type, and monotherapy versus combination therapy.

**Results:**

Across the irAEs evaluated, 41.7% were reported in less than 33% of the trials, 16.6% were reported in 34-66% of trials, and 41.7% were reported in 67-100% of trials. The most frequently reported irAEs included diarrhea/colitis (100%), fatigue (99%), and maculopapular rash (93%). Some infrequently reported irAEs included myocarditis (21%), uveitis (17%), and aseptic meningitis (4%). Additionally, certain organ systems were more frequently reported, including gastroenterology (100%) and endocrine (97%), while others, including cardiology (21%) and ophthalmology (17%), were less frequently reported. The reporting of rarer irAEs significantly increased over time.

**Conclusion:**

Our study demonstrated significant inconsistencies in irAE reporting in the primary literature of trials associated with FDA approvals, particularly for rarer irAEs. Efforts to standardize irAE reporting from clinical trials in the primary literature are needed for more consistent dissemination of information.

## Introduction

The pace of oncology immunotherapy-based Food and Drug Administration (FDA) approvals has been rapid since the first approval of immune checkpoint inhibitors (ICI) in 2011. Specifically, ICI are therapies which induce an antitumor response by blocking innate immune checkpoints. These therapies are monoclonal antibodies which target cytotoxic T-lymphocyte antigen 4 (CTLA-4), programmed cell death 1 (PD-1), or programmed cell death ligand 1 (PD-L1) and reduce tumor-induced T-cell suppression (1, 2). These ICI add an additional pillar to oncology treatment by improving patient outcomes across a variety of tumor types and treatment indications. As ICI have now entered the curative-intent setting and long-term remissions are encountered in a subset of patients with metastatic disease, a better understanding of short and long-term treatment-related toxicity is critical. Still, few studies have assessed how toxicity events are reported in the primary literature.

Predicting immunotherapy response and toxicity have proven challenging given the complex interplay among host, immune system, tumor, microenvironment, and drug (3, 4). Additionally, in contrast to chemotherapy, the timeframe of immune-related adverse events (irAEs) is more unpredictable, although rates of severe adverse events may be lower (5–7). These factors lead to uncertainty regarding the risk and timing of developing irAEs both in patients with metastatic disease treated with palliative intent and in the neoadjuvant and adjuvant settings for curative intent due to the potential for developing chronic irAEs (8). Prior studies demonstrate that the particular choice of ICI (e.g. anti-CTLA-4 vs. anti-PD-1/PD-L1 vs. combination therapy), dose (e.g. ipilimumab 1 mg/kg vs. 3 mg/kg vs. 10 mg/kg), and duration of therapy are all associated with degree of toxicity (5, 7–11).

The presentation of irAEs is broad, ranging from relatively minor toxicities including low-grade rash or pruritus, non-life threatening but potentially permanent toxicities such as thyroid dysfunction, and rare but potentially fatal toxicities including myocarditis, pneumonitis, hepatitis, and neurological issues (3). Each of these irAEs requires clinical expertise for prompt diagnosis and management (3, 12–14). In addition, there are conflicting findings regarding whether irAEs may be associated with improved progression-free survival and overall survival for patients who experience lower-grade toxicity or even multisystem toxicity (15–18). Moreover, the distribution, pattern, and consistency of reporting for irAEs across tumor types and treatment indications have been relatively understudied. Previous studies were more limited in both the breadth of study phases included and the variety of tumor types included in the analyses (19, 20).

The dissemination of scientific information into clinical practice has expanded at a rapid rate via press releases, large volumes of scientific literature, and frequent national and international conferences (21). However, our understanding regarding how toxicity events are captured and reported in the scientific literature, particularly for a rapidly developing field of immuno-oncology with multiple concurrent studies performed across different tumor types, is limited. Monitoring of irAEs for immuno-oncology therapies is critical for providers to understand potential toxicities when implementing therapies into clinical practice and providers often reference the primary literature to understand efficacy and potential toxicity associated with new therapies. Therefore, we sought to evaluate the dissemination and reporting of irAEs in the primary scientific literature.

Herein, we systematically evaluated all pivotal trials leading to FDA approvals of ICI from 2011-2021. We analyzed reporting of irAEs, irAE event rate in the approved trial arm, grade of toxicity, treatment received, monotherapy versus dual inhibitor therapy, and primary tumor type in each trial. The primary objective of the study was to evaluate the consistency and trends of reporting and event rates for irAEs in landmark trials leading to FDA approvals in oncology. In addition, we evaluated differences in reporting for more common versus rare toxicity events, differences based on the organ system of toxicity, and potential changes in reporting of irAEs over time.

## Methods

We examined all ICI FDA approvals from the first approval in 2011 until April 1st, 2021. The data were collected over a 2-year period from November 2022 to November 2024. Institutional Review Board (IRB) approval was waived for this retrospective study given that no patient-protected health information was utilized. The following FDA website which shows publicly available drug approval notifications was used to identify all ICI drug approvals: https://www.fda.gov/drugs/resources-information-approved-drugs/oncology-cancerhematologic-malignancies-approval-notifications.

**Figure 1:**
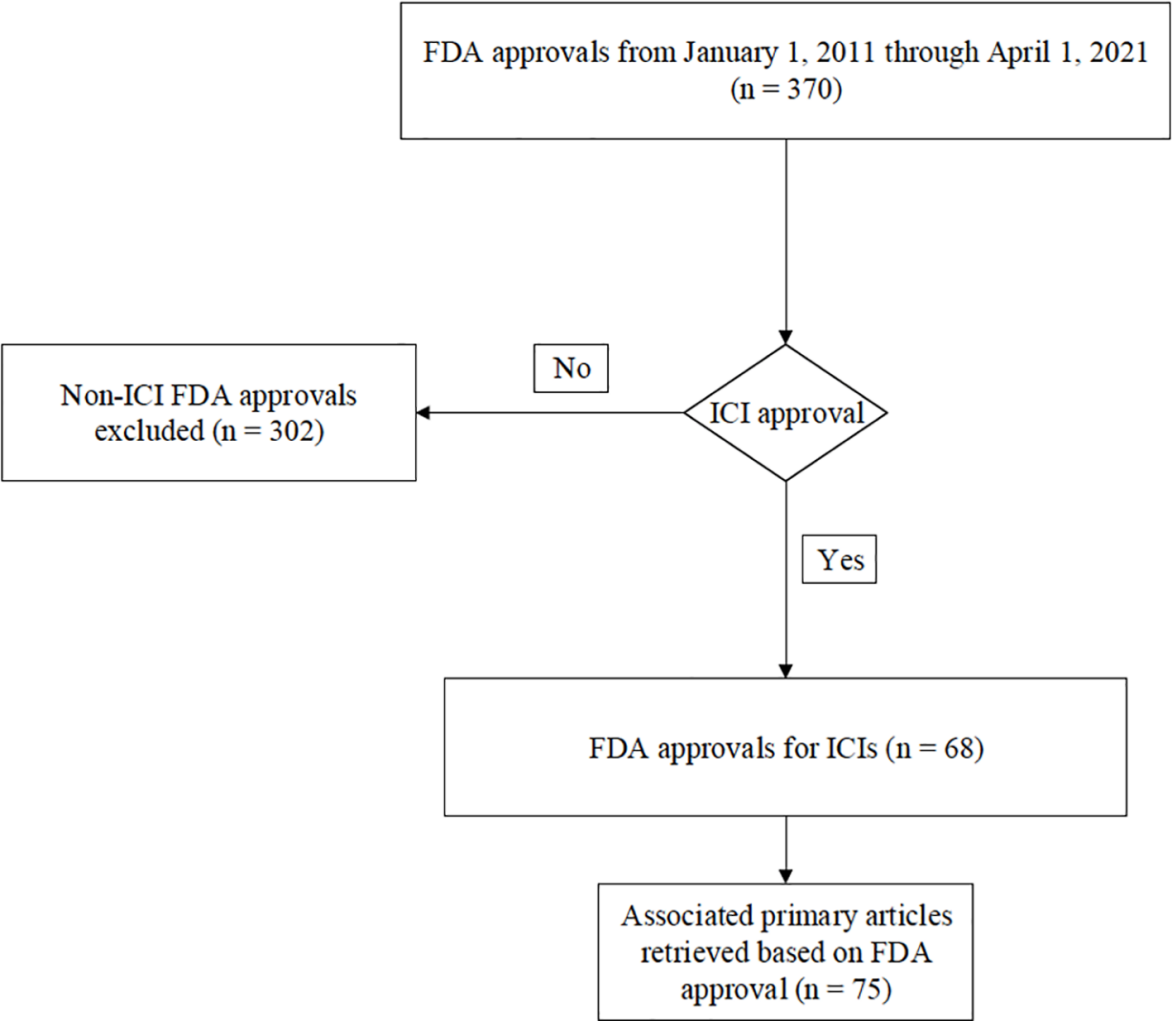
Flow diagram.

There were 68 FDA ICI drug approvals from January 1^st^, 2011, to April 1^st^, 2021 (**Figure 1**). For each approval, the corresponding primary landmark trials were retrieved as referenced in the FDA’s approval notification. There were a total of 75 primary articles that were identified leading to the 68 FDA approvals (**Supplementary Table 1**). For each study, the following data were obtained: publication year, phase of study, primary tumor location, line of therapy, type of immunotherapy administered (e.g., anti-PD-1/anti-PD-L1, anti-CTLA-4, or anti-PD-1/anti-PD-L1 plus anti-CTLA-4), and whether monotherapy versus combination ICI therapy was used. In addition, irAE type, grade of toxicity, and event rate in the trial arm leading to FDA approval were obtained from the primary manuscript, including careful review of all tables, text, and the supplementary appendix of each article. Some FDA approvals were based on more than one study. Our analysis of irAEs reflects whether the individual study reported the adverse event in the trial arm that led to approval. All authors performed the primary review of the manuscripts, and all articles were evaluated by at least two co-authors to ensure fidelity of the data collection. We obtained data regarding the following irAE systems: cardiac, dermatologic, endocrine, general, gastrointestinal, musculoskeletal, rheumatologic, neurologic, ocular, pulmonary, and renal. Toxicities were categorized as “all grade” and “grade 3 or higher.” In total, frequency of reporting regarding 24 different irAEs was obtained based on the classification system utilized in the National Comprehensive Cancer Network (NCCN) Version 1.2024 supportive care guidelines regarding the management of immunotherapy-related toxicities (22). A complete list of the specific irAE data that were collected is shown in **Supplementary Table 2**. In instances where there were discrepancies in irAE terminology or classification across the included trials, the NCCN Version 1.2024 guidelines were referred to for uniformity. For example, some studies may have used different language or classification to describe similar irAEs (ex. transaminitis vs. hepatitis vs. elevated AST/ALT). To resolve this, irAE terminology was mapped from each trial to the relevant terms used in NCCN guidelines. In the above example, based on NCCN Version 1.2024 guidelines, hepatobiliary disorders were considered reported based on the presence of any of those reported adverse events.

Summative data were analyzed using categorical variables. The frequency of irAE reporting and event rate in the primary literature were assessed based on the year of the study, phase of the study, primary tumor type, and ICI type (i.e. monotherapy versus combination therapy). Our primary analysis was based on a binary classification of whether the irAE was reported on a study level (e.g., 1 for yes versus 0 for no). irAEs were analyzed both as individual events (e.g., colitis) and as organ systems-based categories (e.g., gastrointestinal) (**Supplementary Table 3**). Moreover, the percentage of reporting by irAE was divided into three categories: least frequently reported (less than 33%), moderately reported (34-66%), and well reported (67-100%). The trend of reporting (both irAEs and system organs) vs trial characteristics were analyzed by chi-squared tests for trial phase, primary tumor site, and ICI type, and by logistic regression models for publication year.

Additionally, meta-analyses of all grade irAE events in the trial arm leading to FDA approval was conducted by fitting random-effects models on the arcsin square root transformed proportions. For those irAEs that have no event, a continuity correction was applied by adding 0.5 to the zero counts. Between-study heterogeneity was quantified by the I^2^ measure. To further explore possible explanations of this heterogeneity, a meta-regression was conducted using trial and patient characteristics (trial phase, primary tumor site, ICI type, publication year, patient median age, and percent of males as moderators) (**Supplementary Table 4**). The amount of heterogeneity accounted for by these moderators was calculated. The meta-analyses were conducted using the R package “meta” (23).

## Results

### Cohort

In total, there were 75 studies leading to FDA approval included in our analysis from 2011 to April 2021. The pace of approvals accelerated over time with most primary articles published between 2015 and 2021 (**Supplementary Table 5**). The articles included in our analysis were published in the following timeframes: 1 study in 2010, 1 study in 2014, 11 studies in 2015, 9 studies in 2016, 11 studies in 2017, 14 studies in 2018, 10 studies in 2019, 13 studies in 2020, and 5 studies in 2021.

### irAE reporting by phase of study

In our analysis, there were 4 phase I studies, 7 phase I/II studies, 23 phase II studies, 1 phase II/III study, and 40 phase III studies (**Supplementary Table 6**). Of the 24 evaluated irAEs, 11 irAEs (46%) were reported more frequently in phase III studies compared to phase II studies, 9 irAEs (38%) were reported similarly (less than 3% difference) between phase I/II studies and phase III studies, and 4 irAEs (17%) were reported more often in phase I/II studies than phase III studies (**Supplementary Table 7**). Myocarditis, the only cardiac irAE, was more frequently reported in phase III compared to phase I/II studies (32.5% vs 8.6%, p=0.012), and pruritis was more frequently reported in phase I/II compared to phase III studies (97.1% vs 72.5%, p=0.004).

### irAE reporting by tumor type

The FDA approvals included 14 primary tumor sites and 2 tumor agnostic approvals based on microsatellite instability and mismatch repair deficiency. The most common tumor types in the studies included were lung (N=22), skin/cutaneous (N=15), lymphoma (N=6), bladder (N=6), renal (N=5), liver (N=4), head & neck (N=4), esophagus (N=3), breast (N=2), colorectal (N=2), and the following tumor sites with 1 each: cervix, gastric/gastroesophageal junction (GEJ), pleura, and endometrial (**Supplementary Table 8**). We further evaluated the two most common tumor types in terms of number of FDA approvals (lung and skin) for consistency of reporting irAEs. Pneumonitis/interstitial lung disease (ILD) was reported in 100% of lung tumor studies compared with 80% reporting within primary skin cancer studies (p=0.034). Pruritus and uveitis were reported in 100% and 26.7% of skin cancer studies, respectively, which was a significantly higher rate compared to lung tumors with reporting at 68.2% and 0% (p=0.018 and 0.012), respectively, indicating potential differences in reporting based on primary tumor site and type of toxicity (**Supplementary Table 7**).

### irAE reporting by immune checkpoint inhibitor

Of the 24 irAEs evaluated, 11 irAEs (46%) were reported more often in combination therapy compared to monotherapy studies, 3 irAEs (13%) were similarly reported (less than 3% difference), and 10 irAEs (42%) were reported at a greater frequency in monotherapy trials compared to combination therapy studies. Specifically, blistering disorders (16.7% vs 1.5%, p=0.03), elevated lipase (83.3% vs 33.3%, p=0.016) and demyelinating disease (16.7% vs 1.5%, p=0.03) were significantly more frequently reported in combination therapy vs monotherapy studies, and hepatobiliary toxicities (92.8% vs 66.7%, p=0.038) were significantly more frequently reported in monotherapy vs combination therapy studies (**Supplementary Table 7**). For organ systems, endocrine irAEs were significantly more frequently reported in monotherapy vs combination therapy studies (98.6% vs 83.3%, p=0.03).

### irAE reporting by publication year

**Figure 2:**
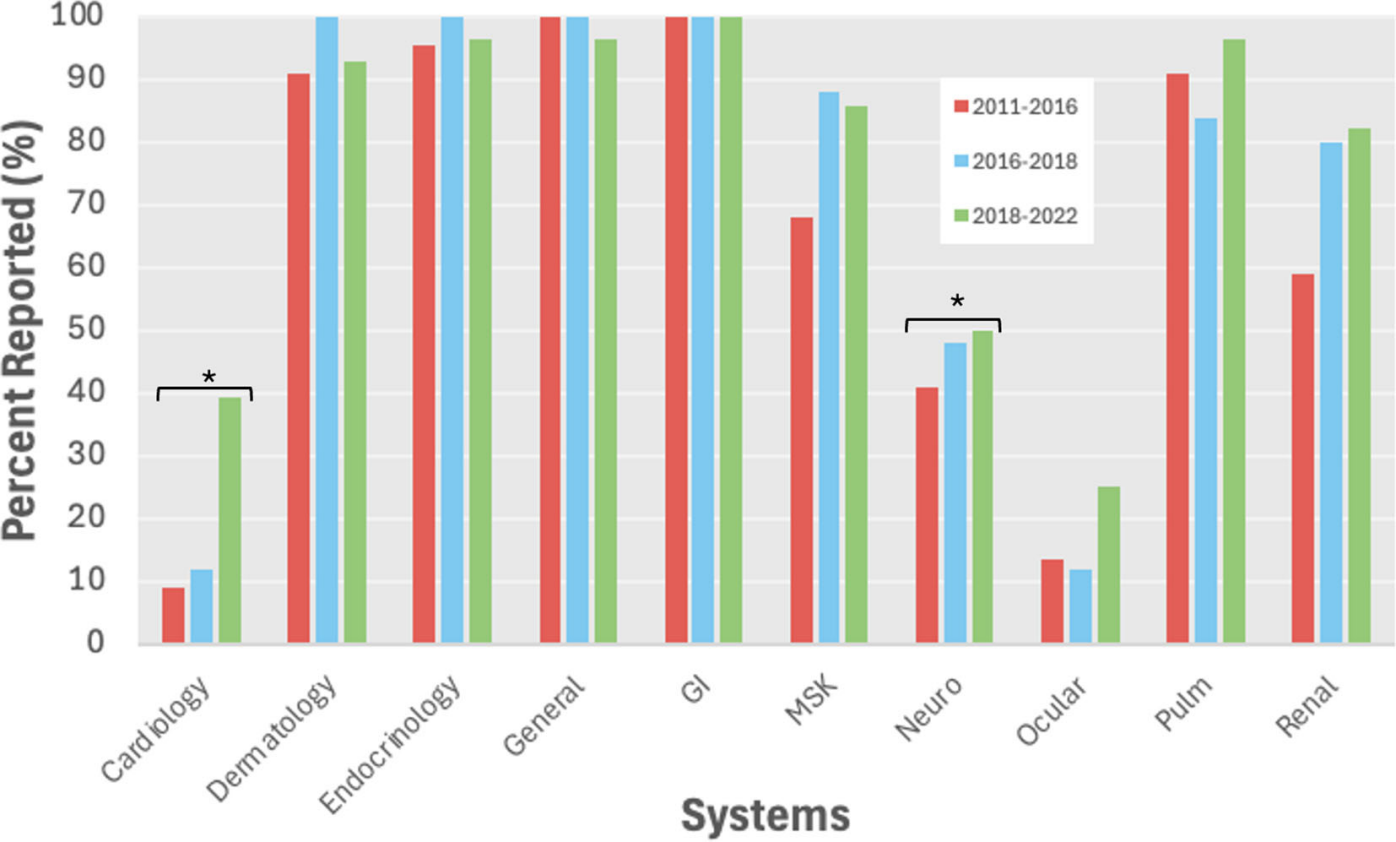
All grade irAE reporting by system over time. *p<0.05. GI, gastrointestinal; MSK, musculoskeletal.

Of the 24 irAEs evaluated, 15 irAEs (62.5%) were reported more often over time, 3 irAEs (13%) were similarly reported (less than 3% difference), 6 irAEs (25%) were similarly reported (yearly odds ratio (OR) between 0.9 - 1.1), and 3 irAEs (12.5%) were reported less often over time. The reporting of certain irAEs significantly increased over time most notably for rarer irAE systems including cardiology (p=0.005) and neurology (p=0.006) (**Figure 2**). Specifically, myocarditis (yearly OR=1.59, p=0.01), pancreatitis (yearly OR=1.49, p=0.005) and nephritis/AKI (yearly OR=1.46, p=0.01) were significantly more likely to be reported over time, while pruritis was significantly less likely to be reported over time (yearly OR=0.66, p=0.031) (**Supplementary Table 7**).

### irAE reporting by organ system

**Figure 3:**
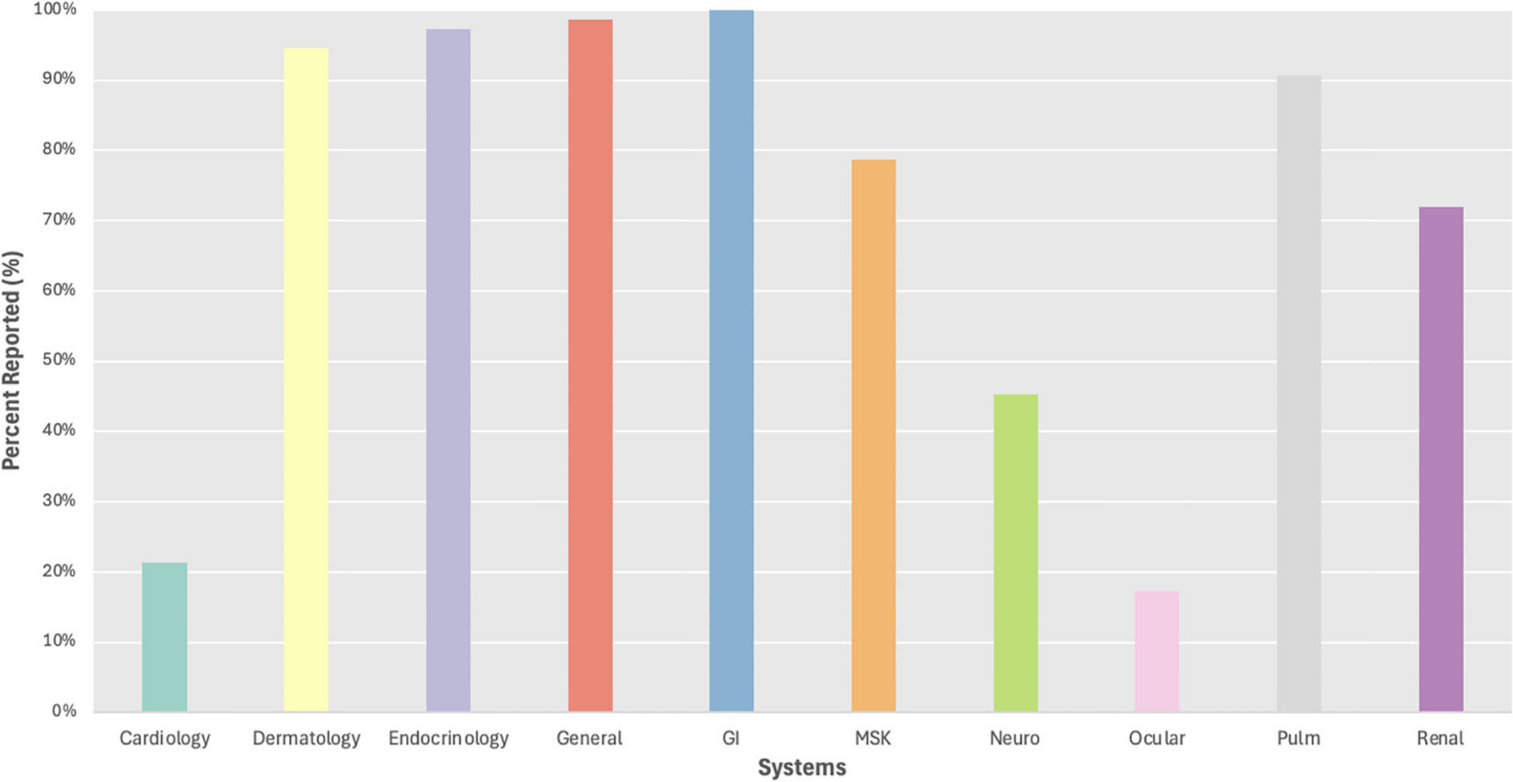
Percent reporting of all grade irAEs. GI, gastrointestinal; MSK, musculoskeletal.

**Figure 4:**
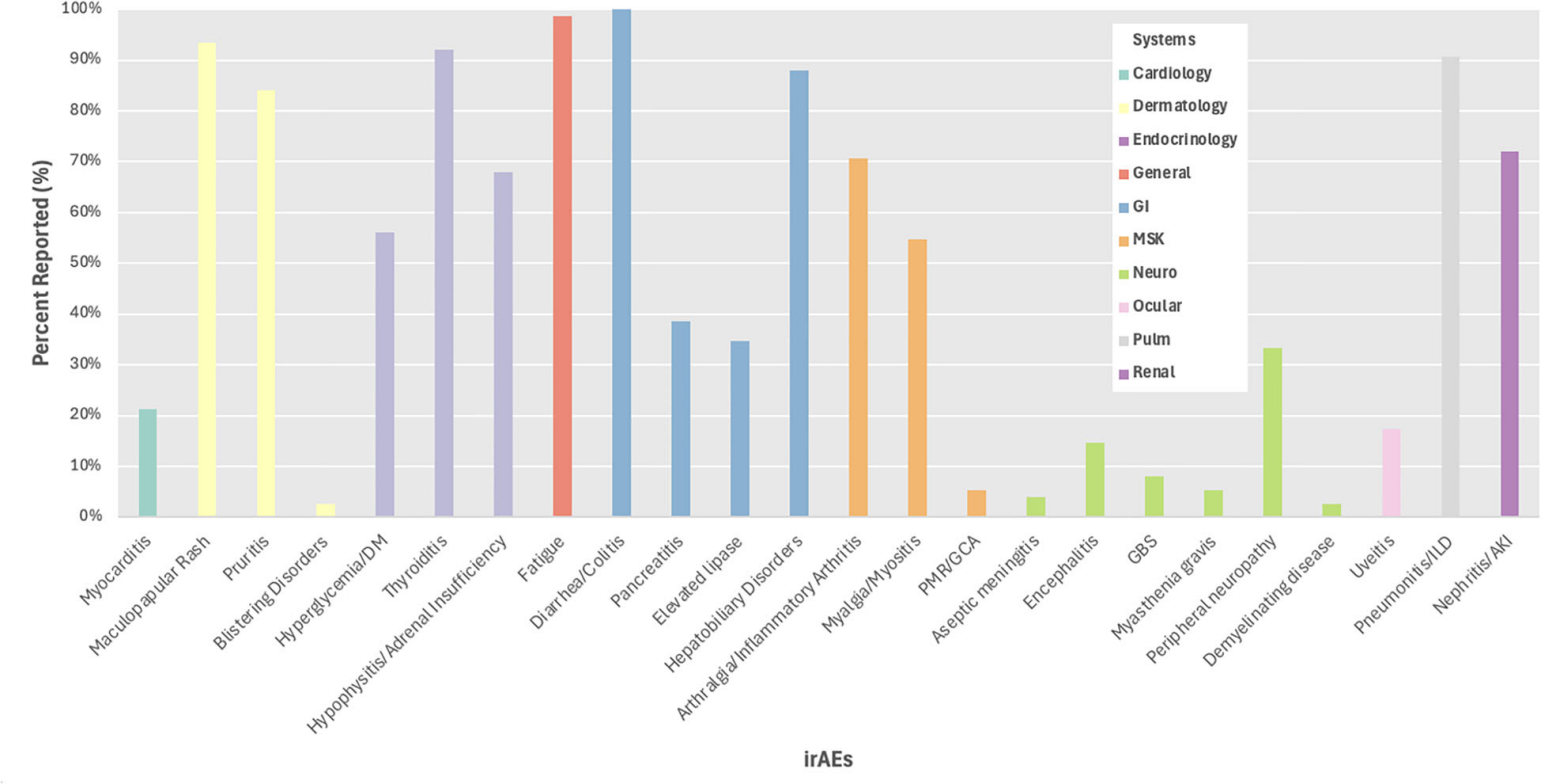
Percent reporting of all grade irAEs by system. DM, diabetes mellitus; PMR, polymyalgia rheumatic; GCA, giant cell arteritis; GBS, Guillain-Barre syndrome; ILD, interstitial lung disease; AKI, acute kidney injury.

We evaluated irAE reporting by organ system and found 3 systems with reporting in less than 50% of trials including ocular (17%), cardiac (21%), and neurological (45%) irAEs, while frequent reporting (>75%) was observed for dermatological, endocrine, general, gastrointestinal, musculoskeletal, and pulmonary irAEs (**Figure 3**). We then evaluated reporting of individual irAEs, which ranged from 3-100% across studies with a mean of 47% and standard deviation of 36 (**Figure 4**). Across the 24 irAEs included, 41.7% were classified as least frequently reported (less than33%), 16.6% were moderately reported (34-66%), and 41.7% were well-reported (67-100%). Examples of least frequently reported irAEs included myocarditis (21%), blistering disorders (3%), encephalitis (15%), peripheral neuropathy (33%), and uveitis (17%). In contrast, the most frequently reported irAEs were maculopapular rash (93%), diarrhea/colitis (100%), hepatobiliary disorders (88%), pneumonitis/ILD (91%), and nephritis/AKI (72%) (**Supplementary Table 7**). There was no significant difference when comparing the reporting of all grade irAEs versus grade 3 or higher irAEs.

### Meta analysis of irAE event rates

The results from the meta-analyses of the event rates for the irAEs are included in **Supplementary Table 9**, where 15 of the 24 irAEs had significant between-study heterogeneity (p<0.05). The study characteristics explain some of the heterogeneity observed. For example, treatment explains more than 20% of the heterogeneity of the rates of maculopapular rash, pruritis, hypophysitis/adrenal insufficiency, and peripheral neuropathy, and tumor site explains more than 20% of the heterogeneity of the rates of thyroiditis, hypophysitis/adrenal insufficiency, fatigue, pancreatitis, elevated lipase, and hepatobiliary toxicities.

## Discussion

Immune checkpoint inhibition is now utilized for patients with metastatic disease across most tumor types, leading to the emergence of immunotherapy-based treatment regimens in the neoadjuvant and adjuvant settings. Given this expansion of immunotherapy use, guidelines have been developed to address the management of irAEs (13, 24, 25). For these reasons, adequate understanding of short and long-term irAEs, including some rare and potentially life-threatening toxicities, is essential. Despite the requirement to submit safety and toxicity data to the FDA for a novel drug indication, the primary basis for dissemination of trial information to providers, including efficacy and potential toxicity of novel therapeutics, is via reporting in primary manuscripts in peer-reviewed medical journals and at medical conferences. Therefore, we examined the reliability and consistency of reporting irAEs in the primary literature across all FDA approvals of ICI over a ten-year period. To our knowledge, this is the first study to systematically evaluate reporting of irAEs in phase I to III trials leading to FDA approvals of ICI, including anti-PD-1/anti-PD-L1, anti-CTLA-4, or anti-PD-1/anti-PD-L1 plus anti-CTLA-4, via an organ-systems based approach. This study has important implications for understanding irAEs across tumor types and how these toxicities are reported in landmark studies leading to FDA approval.

Our analysis found inconsistent reporting of irAEs across landmark clinical trials, particularly for rarer irAEs. We found significant discrepancies in reporting across different tumor types despite the use of the same ICIs and across different phases of drug development (e.g., Phase I, II, and III). For example, rarer irAEs such as myocarditis, uveitis, and aseptic meningitis were reported in only 21%, 17%, and 4%, of studies, respectively, whereas more common irAEs such as thyroiditis, diarrhea/colitis, and pneumonitis/ILD were reported in 92%, 100%, and 91% of the trials, respectively. Approximately 70% of the least frequently reported irAEs were reported at higher frequency in Phase III trials compared to Phase I/II trials, a finding that may have been observed due to the relatively smaller sample sizes in Phase I/II studies. Interestingly, our data demonstrated that the reporting of rarer irAEs in the literature increased over time, suggesting potential improved awareness of less common irAEs over time.

Additionally, we found variability of irAE reporting by organ systems. Organ systems that were reported in greater than 67% of studies included dermatology, endocrinology, general, gastroenterology, musculoskeletal, pulmonology, and renal (**Figure 1**). Within the most frequently reported organ systems (e.g., dermatologic), we observed that more common irAEs, such as maculopapular rash was reported in 93% of trials, while rarer irAEs such as blistering disorders were reported in only 3% of studies, and this varied based on tumor type with dermatologic irAEs reported more often in studies of primary skin tumors compared to lung tumors. Similarly, endocrine as a system was well-reported, but this primarily represented the reporting of thyroiditis (92%), while other endocrine irAEs were less frequently reported including hyperglycemia/DM (56%). We were unable to distinguish between events not occurring (e.g. 0%) versus lack of reporting with uncertain frequency of occurrence, which may have contributed to underreporting of rarer irAEs. Finally, our meta-analysis demonstrated some differences in incidence of certain irAEs by primary tumor site, but these results should be viewed with caution given the heterogeneity of the studies included in our analysis.

Previous studies have also assessed the reporting of irAEs. A prior systematic review of irAE reporting including 50 trials found that the median rate of grade 3 or 4 adverse events was 21% with lack of reporting regarding timeframe, management, and reversibility of these events (19). Additionally, another systematic review which evaluated the harm reporting quality of 123 phase II and III immuno-oncology trials found that the methodological aspects of irAE data collection were poorly reported (3%) as well (20). Similarly, another review evaluated thoracic oncology clinical trials presented at the American Society for Clinical Oncology annual meetings between 2017 and 2019, detailing inconsistent information presented to attendees (26). For example, the threshold at which adverse events were reported was highly variable across trials, ranging from reporting adverse events for only one affected patient to greater than 40% incidence among patients (26). Our study expands the existing literature on the reporting frequency of irAEs in immune checkpoint inhibitor studies by including phase I-III trials and multiple primary tumor types and exclusively focuses on the primary literature of studies that led to FDA approval of the checkpoint inhibitor.

Through the years, multiple mechanisms to enhance reporting have been evaluated including systems-based approaches and online self-reporting tools to evaluate patient toxicity (27, 28). Given the somewhat unpredictable timeframe and severity of irAEs, this presents a challenge for reliable dissemination of information, particularly as these therapies have expanded to the neoadjuvant and adjuvant settings. In fact, lack of awareness and vigilance to recognize irAEs may present a clinical challenge leading to a delay in diagnosis and treatment. Thus, efforts should be made not only to standardize a method of reporting adverse events in the literature, but also for continued monitoring through real-world data. Real-world cohort toxicity has been noted to be higher than trial toxicity. For example, in KEYNOTE-024, hypophysitis was reported in 0.6% of patients receiving pembrolizumab (29). However, in a large commercial insurance claims database, 2.4% of non-small cell lung cancer patients receiving pembrolizumab were diagnosed with hypophysitis (30). Efforts to improve clinical trial reporting have been initiated, including convening multidisciplinary working groups including medical oncologists, immunologists, statisticians, industry, and government stakeholders (31).

Our study has several limitations. First, within the trials, there was inconsistency in the language used to report various irAEs and the attribution of causality (immune-related versus non-immune related), which may have led to variability in reporting of adverse events. There was more than one Common Terminology Criteria for Adverse Events (CTCAE) version used in the included trials from 2011–2021 which differed in terminology and grading. Similarly, the variation of irAE reporting requirements across different journals limits standardization of reporting across studies in the literature. Second, we were not able to distinguish between irAEs that did not occur versus those that were not reported. This may have led to a greater tendency for rarer irAEs to be labeled as “not reported.” Third, in many cases, these studies had a limited follow up period prior to publication. Real-world data indicate potential higher toxicity in clinical practice compared to patients participating in clinical trials, and therefore the timing of certain later-onset irAEs was challenging to capture (29, 30). Fourth, publication bias likely contributed to our findings and can lead to an underestimation of the frequency and severity of irAEs in the included study as those more favorable results are more likely to be published.

In conclusion, our findings demonstrated that the reporting of irAEs in the primary literature leading to FDA approvals was inconsistent. Standardized reporting of irAEs in the primary literature is crucial as it serves as a key resource for both clinical providers and patients when evaluating and managing therapies. Amongst a wide variety of primary tumor types and immunotherapy regimens, rarer irAEs were generally less frequently reported, and more common irAEs were more likely to be reported in the primary literature. Efforts to standardize terminology and structure of adverse event reporting including instituting consistent reporting requirements across journals would ensure more transparent and comparable data interpretation across clinical trials (**Supplementary Table 10**). Overall, more consistent reporting of adverse events in the primary literature of landmark trials would provide clinicians with transparent dissemination of information enabling a more precise risk-benefit evaluation for patients and clinical providers.

## Data Availability

The original contributions presented in the study are included in the article/**Supplementary Material**. Further inquiries can be directed to the corresponding author.
