# Supplementary material for: Reporting of immune-related adverse events in US Food and Drug Administration approvals of immune checkpoint inhibitors
Source: Front Oncol. 2025 Aug 13;15:1606599. doi: 10.3389/fonc.2025.1606599 (PMC12380579; doi:10.3389/fonc.2025.1606599)
Supplement: Supplementary file 1 [file DataSheet1.docx]

Supplemental Table 1: Studies leading to FDA approvals

| **Study leading to approval** | **Year of study** |
| --- | --- |
| Ipilimumab in patients with metastatic melanoma | 2010 |
| Pembrolizumab in ipilimumab-refractory advanced melanoma | 2014 |
| EORTC 18071 | 2015 |
| Nivolumab and ipilimumab versus ipilimumab in untreated melanoma | 2015 |
| Nivolumab for relapsed or refractory Hodgkin's lymphoma | 2015 |
| CheckMate 063 | 2015 |
| Nivolumab in advanced renal cell carcinoma | 2015 |
| Nivolumab in non-small cell lung cancer (NSCLC) | 2015 |
| CheckMate 037 | 2015 |
| KEYNOTE-001 | 2015 |
| KEYNOTE-002 | 2015 |
| KEYNOTE-010 | 2015 |
| KEYNOTE-006 | 2015 |
| Atezolizumab in patients with locally advanced and metastatic urothelial carcinoma | 2016 |
| POPLAR Study | 2016 |
| OAK study | 2016 |
| CheckMate 032 | 2016 |
| Nivolumab for classical Hodgkin's lymphoma | 2016 |
| Nivolumab for recurrent squamous cell carcinoma of the head and neck | 2016 |
| KEYNOTE-021 | 2016 |
| Pembrolizumab in advanced Merkel cell carcinoma | 2016 |
| Pembrolizumab for PD-L1-Positive NSCLC | 2016 |
| Durvalumab in locally advance or metastatic urothelial cancer | 2017 |
| PACIFIC study | 2017 |
| CheckMate 040e | 2017 |
| CheckMate 275 | 2017 |
| CheckMate 142 | 2017 |
| Nivolumab for relapsed or progressive classical Hodgkin's lymphoma | 2017 |
| CheckMate 238 | 2017 |
| Pembrolizumab for platinum and cetuximab refractory head and neck cancer | 2017 |
| Pembrolizumab in relapsed/ refractory classic Hodgkin's lymphoma | 2017 |
| Mismatch repair deficiency predicts response of solid tumors to PD-1 blockade | 2017 |
| KEYNOTE-045 | 2017 |
| IMpassion130 | 2018 |
| IMpower150 | 2018 |
| IMpower133 | 2018 |
| JAVELIN Solid Tumor | 2018 |
| JAVELIN Merkel 200 | 2018 |
| Cemiplimab in advanced cutaneous squamous-cell carcinoma | 2018 |
| CheckMate 214 | 2018 |
| CheckMate 205 | 2018 |
| KEYNOTE 180 | 2018 |
| KEYNOTE-059 | 2018 |
| KEYNOTE-224 | 2018 |
| KEYNOTE-407 | 2018 |
| KEYNOTE-189 | 2018 |
| Adjuvant pembrolizumab in resected stage III melanoma | 2018 |
| IMPower 130 | 2019 |
| JAVELIN Renal 101 | 2019 |
| CASPIAN | 2019 |
| CheckMate 227 | 2019 |
| ATTRACTION-3 | 2019 |
| KEYNOTE-158 | 2019 |
| KEYNOTE-158 | 2019 |
| KEYNOTE-426 | 2019 |
| KEYNOTE-042 | 2019 |
| KEYNOTE-017 | 2019 |
| IMBrave 150 | 2020 |
| IMPower 110 | 2020 |
| IMspire150 | 2020 |
| JAVELIN BLADDER 100 | 2020 |
| CheckMate 040 | 2020 |
| KEYNOTE 146 - Study 111 | 2020 |
| KEYNOTE-629 | 2020 |
| KEYNOTE-355 | 2020 |
| KEYNOTE-177 | 2020 |
| KEYNOTE-181 | 2020 |
| KEYNOTE-048 | 2020 |
| KEYNOTE-048 | 2020 |
| KEYNOTE 158 and 028 | 2020 |
| EMPOWER-Lung 1 | 2021 |
| CheckMate 9LA | 2021 |
| CheckMate 743 | 2021 |
| KEYNOTE-9ER | 2021 |
| KEYNOTE-204 | 2021 |

Supplemental Table 2: irAEs by system

| **System** | **irAE** |
| --- | --- |
| Cardiology | Myocarditis |
| Dermatology | Maculopapular Rash |
|  | Pruritis |
|  | Blistering Disorders |
| Endocrinology | Hyperglycemia/Diabetes mellitus |
|  | Thyroiditis |
|  | Hypophysitis/Adrenal Insufficiency |
| General | Fatigue |
| Gastroenterology | Diarrhea/Colitis |
|  | Pancreatitis |
|  | Elevated lipase |
|  | Hepatobiliary Disorders |
| Musculoskeletal | Arthralgia/Inflammatory Arthritis |
|  | Myalgia/Myositis |
|  | Polymyalgia rheumatica/Giant cell arteritis |
| Neurology | Aseptic Meningitis |
|  | Encephalitis |
|  | Guillain-Barre Syndrome |
|  | Myasthenia gravis |
|  | Peripheral neuropathy |
|  | Demyelinating disease |
| Ocular | Uveitis |
| Pulmonary | Pneumonitis/Interstitial lung disease |
| Nephrology | Acute kidney injury/Nephritis |

Supplemental Table 3: Reporting of irAEs by study


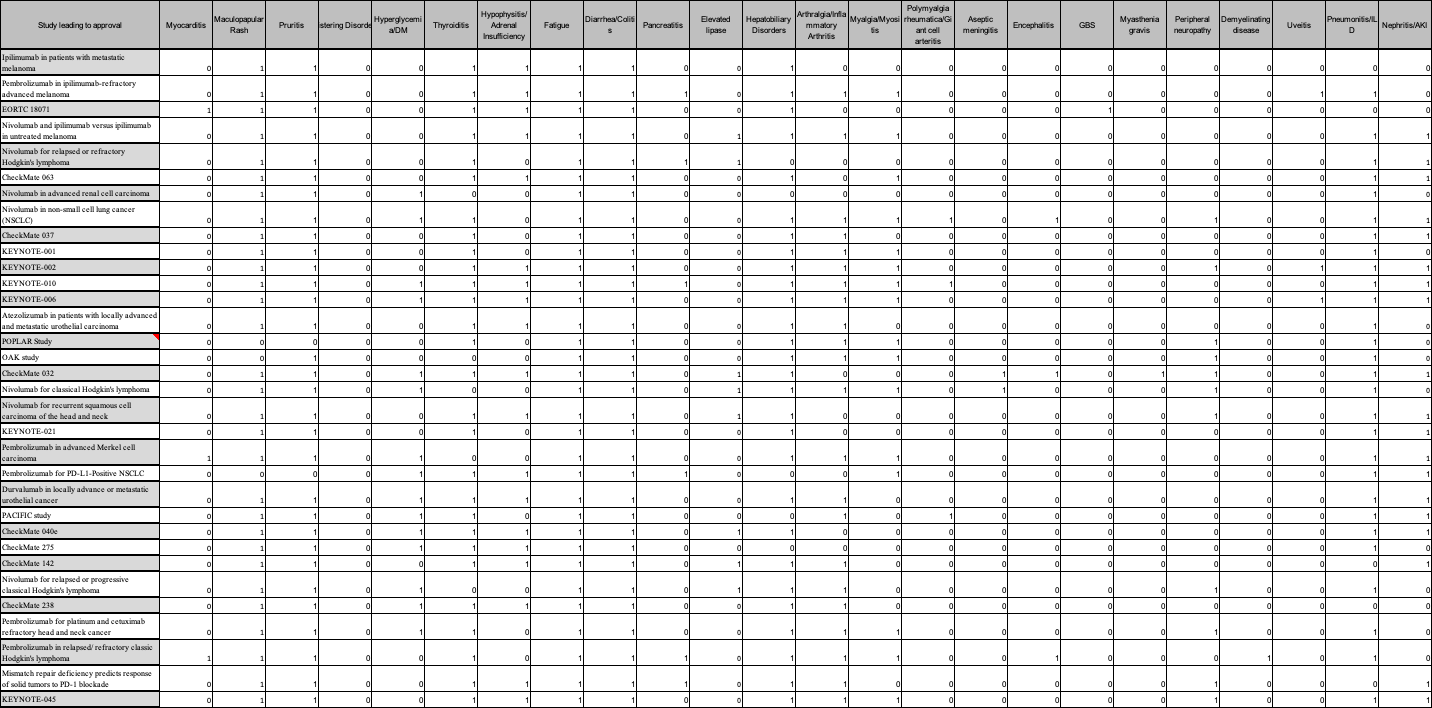


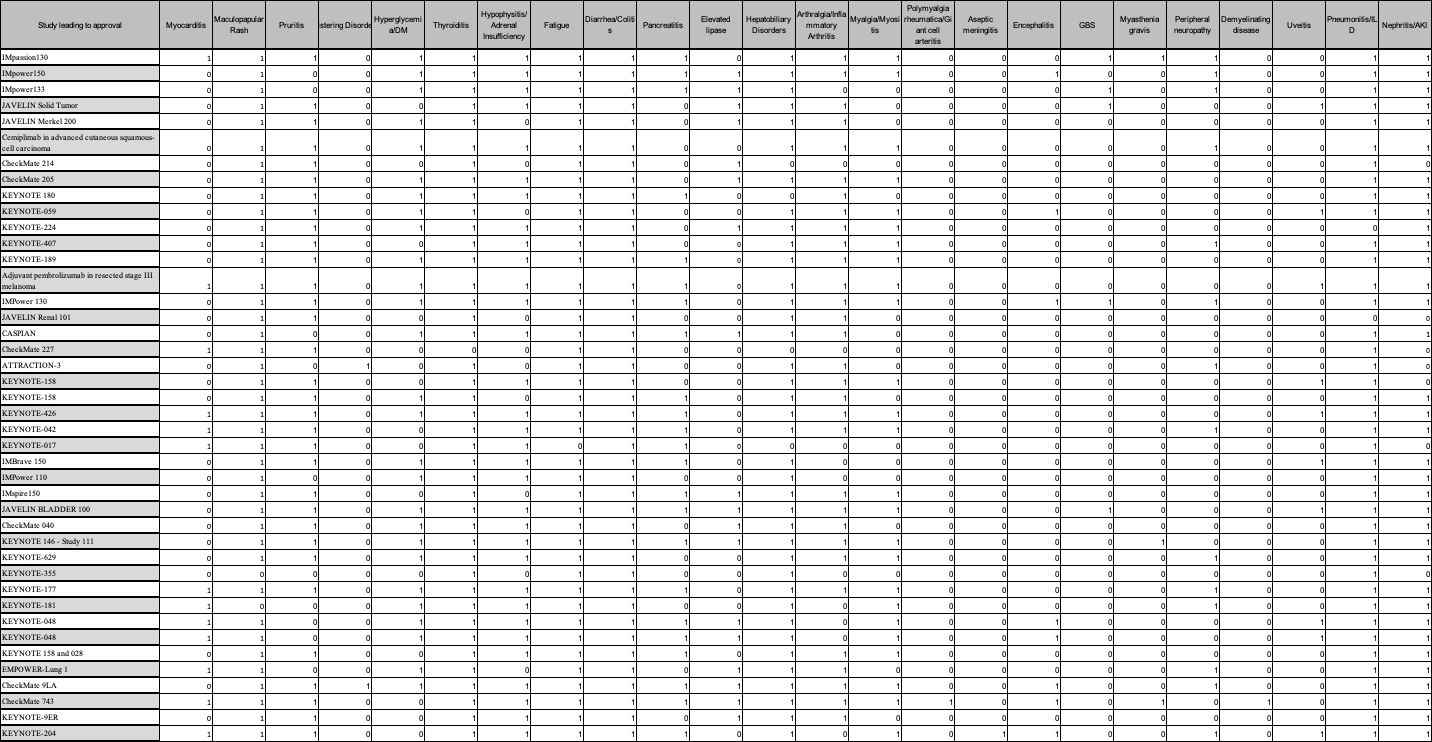


0 indicates not reported. 1 indicates reported.

Supplemental Table 4: Patient demographics


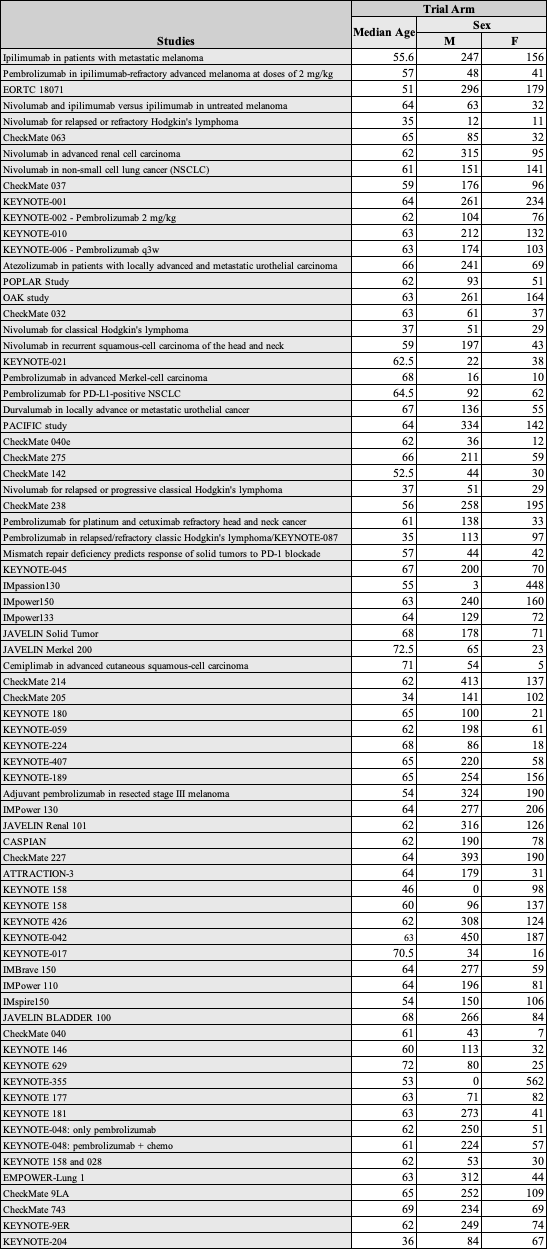


Supplemental Table 5: Number of studies by year

| Year | 2010 | 2014 | 2015 | 2016 | 2017 | 2018 | 2019 | 2020 | 2021 |
| --- | --- | --- | --- | --- | --- | --- | --- | --- | --- |
| Number of studies | 1 | 1 | 11 | 9 | 11 | 14 | 10 | 13 | 5 |

Supplemental Table 6: Number of studies by phase

| **Study Phase** | **Number of Studies** |
| --- | --- |
| I | 4 |
| I, II | 7 |
| II | 23 |
| II, III | 1 |
| III | 40 |

Supplemental Table 7: Overall reporting of irAEs by trial characteristics


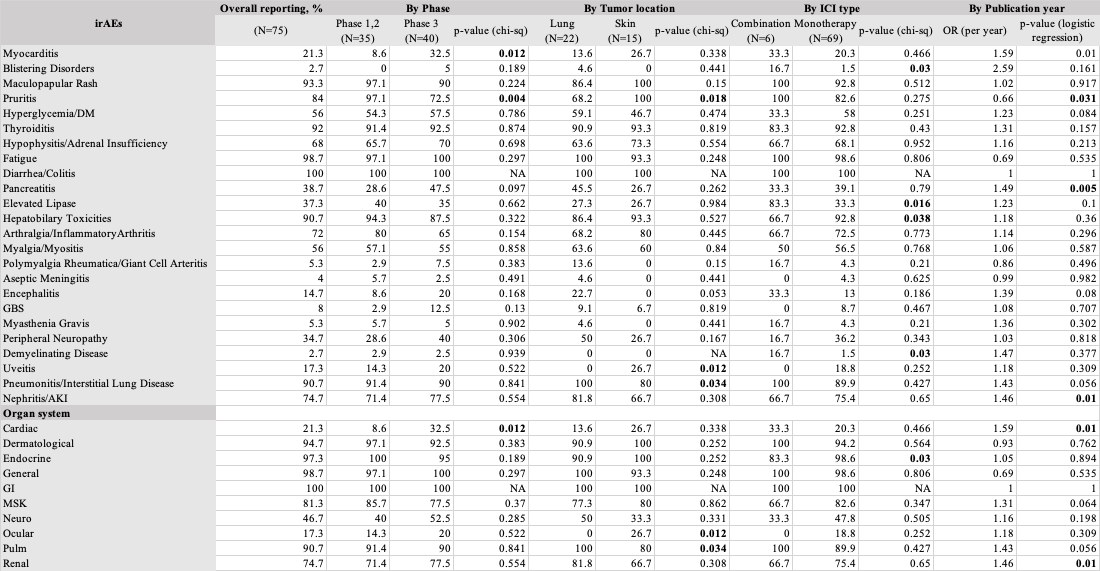


Supplemental Table 8: Number of studies by phase for each primary tumor site

| **Primary tumor site** | **Phase I** | **Phase I, II** | **Phase II** | **Phase II, III** | **Phase III** |
| --- | --- | --- | --- | --- | --- |
| Lung | 1 | 2 | 3 | 1 | 15 |
| Skin | 1 | 1 | 6 | 0 | 7 |
| Lymphoma | 1 | 0 | 4 | 0 | 1 |
| Bladder | 1 | 1 | 2 | 0 | 2 |
| Kidney | 0 | 0 | 0 | 0 | 5 |
| Liver | 0 | 2 | 1 | 0 | 1 |
| Head and neck | 0 | 0 | 1 | 0 | 3 |
| Esophageal | 0 | 0 | 1 | 0 | 2 |
| Breast | 0 | 0 | 0 | 0 | 2 |
| Colorectal | 0 | 0 | 1 | 0 | 1 |
| Cervical | 0 | 0 | 1 | 0 | 0 |
| Pleural | 0 | 0 | 0 | 0 | 1 |
| GEJ | 0 | 0 | 1 | 0 | 0 |
| Endometrial | 0 | 1 | 0 | 0 | 0 |
| Tumor agnostic | 0 | 0 | 2 | 0 | 0 |

Supplemental Table 9: Meta-analysis of event rate of irAEs by trial and patient characteristics


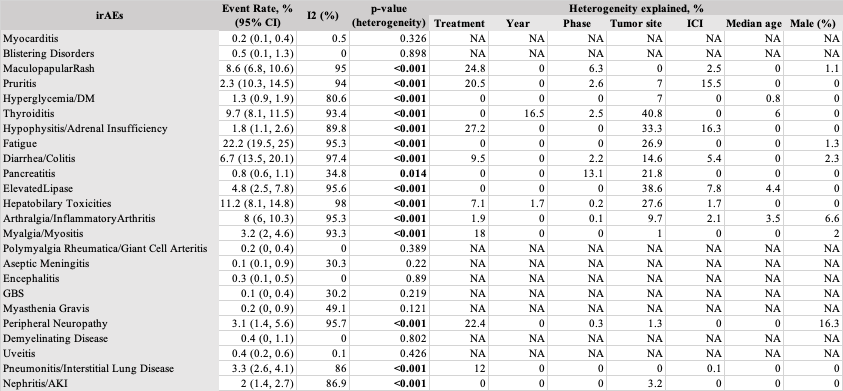


Supplemental Table 10: Proposed comprehensive reporting framework

| irAEs | Grade 1 | Grade 2 | Grade 3 | Grade 4 | Grade 5 |
| --- | --- | --- | --- | --- | --- |
| Cardiology |  |  |  |  |  |
| Myocarditis |  |  |  |  |  |
| Dermatology |  |  |  |  |  |
| Maculopapular Rash |  |  |  |  |  |
| Pruritis |  |  |  |  |  |
| Blistering Disorders |  |  |  |  |  |
| Endocrinology |  |  |  |  |  |
| Hyperglycemia/DM |  |  |  |  |  |
| Thyroiditis |  |  |  |  |  |
| Hypophysitis/  Adrenal Insufficiency |  |  |  |  |  |
| Gastroenterology |  |  |  |  |  |
| Diarrhea/Colitis |  |  |  |  |  |
| Pancreatitis |  |  |  |  |  |
| Elevated lipase |  |  |  |  |  |
| Hepatobiliary disorders |  |  |  |  |  |
| General |  |  |  |  |  |
| Fatigue |  |  |  |  |  |
| Musculoskeletal |  |  |  |  |  |
| Arthralgia/  Inflammatory Arthritis |  |  |  |  |  |
| Myalgia/Myositis |  |  |  |  |  |
| Polymyalgia rheumatica/  Giant cell arteritis |  |  |  |  |  |
| Nephrology |  |  |  |  |  |
| AKI/Nephritis |  |  |  |  |  |
| Neurology |  |  |  |  |  |
| Aseptic meningitis |  |  |  |  |  |
| Demyelinating disease |  |  |  |  |  |
| Encephalitis |  |  |  |  |  |
| GBS |  |  |  |  |  |
| Myasthenia gravis |  |  |  |  |  |
| Peripheral neuropathy |  |  |  |  |  |
| Ophthalmology |  |  |  |  |  |
| Uveitis |  |  |  |  |  |
| Pulmonology |  |  |  |  |  |
| Pneumonitis/ILD |  |  |  |  |  |

Supplemental Figure 1: All grade irAE reporting by phase

Supplemental Figure 2: Heat map of irAE reporting by system and primary tumor site
